# Link of dietary patterns with metabolic syndrome: analysis of the National Health and Nutrition Examination Survey

**DOI:** 10.1038/nutd.2017.11

**Published:** 2017-03-20

**Authors:** M Mazidi, S Pennathur, F Afshinnia

**Affiliations:** 1 Key State Laboratory of Molecular Developmental Biology, Institute of Genetics and Developmental Biology, Chinese Academy of Sciences, Beijing, China; 2 Institute of Genetics and Developmental Biology, International College, University of Chinese Academy of Science, Beijing, China; 3 Division of Nephrology, Department of Internal Medicine, University of Michigan, Ann Arbor, MI, USA

## Abstract

**Background:**

Population-based interventions aimed at halting the increasing prevalence of metabolic syndrome (MetS) require thorough understanding of dietary interplays. Objective is to identify the independent dietary nutrients associated with MetS and its components using dietary pattern identification and the single-nutrient approaches in The United States.

**Methods:**

This is a cross-sectional observation. Participants are selected from the National Health and Nutrition Examination Survey (NHANES) with available dietary intake, biochemical and anthropometrical data from 2001 to 2012. Exposure is diet obtained from 24-h dietary recall. Main outcome measure is MetS and its components.

**Results:**

Overall, 23 157 eligible individuals including 6561 with MetS were included in the final analysis. Using principle component analysis, we identified three food patterns that explained 50.8% of the variance of the dietary nutrient consumption. The highest quartile of the factor score representative of saturated/monounsaturated fatty acids or the first dietary pattern was associated with 1.27-fold (95% confidence interval (CI): 1.10–1.46, *P*=0.001) higher odds of association with MetS when compared with the first quartile. The second pattern representative of vitamins and trace elements had an odds ratio of 0.79 (95% CI: 0.70–0.89, *P*<0.001) for association with MetS, and the third pattern representative of polyunsaturated fatty acids did not have any association with MetS. The nutrient-by-nutrient approach showed that mild alcohol intake and lower consumption of total saturated fatty acids and sodium were associated with lower risk of MetS.

**Conclusions:**

Application of multiple complementary analytic approaches reveals more comprehensive dietary determinants of MetS and its components as potential intervening targets.

## Introduction

Metabolic syndrome (MetS) is a major public health issue worldwide with increased risk of type 2 diabetes, cardiovascular morbidity and mortality.^1, 2^ Its prevalence is increasing rapidly, likely due to changes in lifestyle factors, socioeconomic status and dietary habits.^3, 4^ There is eminent need for population-based interventions to halt the rapidly increasing prevalence of MetS. For such interventions to be effective, a thorough understanding of the dietary interplays of MetS and its components is crucial. Several studies have tried to shed light on the dietary determinant of MetS.^5, 6, 7, 8, 9, 10, 11, 12^ Some of their limitations include relatively small sample size of the studies,^5, 7, 8, 10^ lack of diversity of the populations,^12^ limitation in generalizability of the findings^12^ and single-nutrient approach,^6, 7, 8, 9, 10, 11^ whereas diet by definition is complex. As a result of more recognition of limitations inherent to the single-nutrient approach, dietary pattern analysis has emerged as an attractive alternative approach for examining the effect of overall diet reflecting the real-world eating behaviors of the population.^13^ In dietary pattern analysis, application of data reduction techniques such as principle component analysis (PCA) allows identification of groups of nutrients by creation of secondary variables representative of nutrients that often times are consumed together. Such quantitative secondary variables representative of distinct dietary patterns can be used in downstream analysis to explore the links between the specific dietary patterns and MetS. However, this later approach is also limited by its failure to detect the predictive power of significant single nutrients, which may not group together along with the other complex-dietary patterns. To our knowledge, there is no study that has applied both approaches as complementary tools to illuminate a comprehensive view of dietary interplays of MetS and its components. The aims of this study are as follows: (a) to identify the dietary patterns in the United States general adult population using the NHANES data set; (b) to compare risk of MetS and its components by quartiles of the secondary variables representative of the dietary patterns; and (c) to estimate the risk of MetS and its components using a nutrient-by-nutrient approach. We hypothesize that risk predictive models derived from analysis of dietary patterns are different from those obtained from the nutrient-by-nutrient approach, and therefore the application of both approaches may provide a more comprehensive view on dietary determinants of Mets.

## Materials and methods

### Study population

This is a cross-sectional study using the publically available National Health and Nutrition Examination Survey (NHANES) data set. NHANES is a nationally representative sample of the United States and an ongoing survey with details available elsewhere.^14, 15, 16, 17^ For the data collection and physical examination of the NHANES, informed consent was obtained from all adult participants, and the National Center for Health Statistics Research Ethics Review Board approved the protocol. As the data are publically available, the study was exempt from obtaining additional local institutional review board approval. We applied six 2-year NHANES survey cycles from 2001 to 2012. Inclusion criteria were age of 18 years and older, in participants with available demographic, laboratory and nutrients derived from day 1 dietary recall questionnaire (Supplementary Data). Participants with unavailable laboratory data or those who did not respond to dietary recall questionnaire were excluded. The details of dietary interviews have been presented elsewhere.^18^ In brief, the interviews were in person and conducted by trained qualified personnel in the mobile examination centers with the application of computerized Dietary Recall Interview and Data Processing Systems aimed at computing total nutrient intakes from the consumed foods and dietary supplements. Rationale for using dietary recall from day 1 and not day 2 was higher likelihood of accuracy of recall on more recent events. Overall, we identified 23 157 eligible participants aged 18 years or older for the downstream analyses.

### Metabolic syndrome

We used the National Cholesterol Education Program's Adult Treatment Panel III report to define MetS,^19^ if at least three of the following five criteria were satisfied: (1) waist circumference ⩾102 cm in men or ⩾88 cm in women; (2) triglycerides ⩾150 mg dl^−1^; (3) high-density lipid (HDL) cholesterol <40 mg dl^−1^ in men or <50 mg dl^−1^ in women; (4) systolic blood pressure ⩾130 or diastolic blood pressure ⩾85 mm Hg; (5) fasting blood glucose ⩾100 mg dl^−1^.

### Statistical analysis

We conducted the analyses according to the guidelines recommended by the Centers for Disease Control for analysis of complex NHANES data set accounting for the masked variance and using the proposed weighting methodology.^20^ We applied two complementary analytic approaches. In the first approach, we used principle component (PC) factor analysis with Varimax orthogonal transformation to generate PCs representative of dietary patterns based on the highest correlation coefficients between the nutrients constructing each PC.^21^ All the necessary prerequisites of PC analysis including linearity, Kaiser–Meyer–Olkin measure of 0.88, and the significant Bartlett's test of sphericity (*P*<0.001) were met. We used an Eigen value of >4 to obtain maximum number of interpretable dietary patterns that covered over 50% of the total variance of dietary intake. We then used regression method to calculate the factor scores of each nutrient pattern for each study participants.^22^ Then, we categorized the nutrient patterns into subgroups based on quartiles of their corresponding factor scores. We applied age-, sex- and race-adjusted analysis of covariance to compare the nutrient intake across quartiles of dietary patterns (Table 2). To identify dietary predictors of Mets and its components, we subsequently applied logistic regression analysis and estimated the age-, sex- and race-adjusted odds of Mets (and its components) for each quartile of dietary patterns compared with the fourth quartile as reference category. We performed 1000 simulation per sample using bootstrapping method and noted no alteration in the results, supporting the stability of the PCs. In the second approach, we compared age-, gender- and race-adjusted mean of each nutrient by categories of MetS and its components using analysis of covariance. We ranked the list of nutrient by their nominal significance (*P*<0.05). Then, we used logistic regression analysis with backward deletion of non-significant nutrients to identify the nutrients independently associated with MetS and its components. Given the 63 examined nutrients, the Bonferroni-adjusted *P*-value used for multiple comparisons in logistic regression models was calculated to be 0.00079 (0.05/63), which was used as the cutoff for inclusion of a nutrient in the logistic regression model. Statistical analysis was performed using SPSS version 18.0 (SPSS Inc., Chicago, IL, USA).

## Results

### Description of population

Table 1 shows the distribution of baseline demographics by status of MetS. Overall, 23 157 eligible individuals including 6561 with and 16 596 without MetS were selected from the publically available NHANES data set were included in the final analysis. There was no significant association between gender and MetS. Overall, individuals with MetS were older (*P*<0.001). The distribution of MetS was also significantly different by race, so that those in the non-Hispanic white race category were more likely, but those in the non-Hispanic black race category were less likely to have had MetS (*P*<0.001). Similarly, all five components of MetS were more prevalent in participants with Mets (*P*<0.001).

### Data reduction approach

Using a PCA method, we reduced the dietary variables from 63 variables to three total PCs that altogether explained 50.8% of the variance of the dietary nutrient consumption. Table 2 illustrates the nutrients that contributed to each PC. Accordingly, the first PC was mainly representative of saturated and monounsaturated fatty acids, although consumption of carbohydrates was also associated with this PC; the second PC represented vitamins and trace elements, and the third PC was mainly representative of polyunsaturated fatty acids (PUFA). Table 2 additionally showed the age-, gender- and race-adjusted mean of each nutrient by quartiles of the three PCs. Accordingly, for the nutrients that were constituent elements of a PC, there were highly statistically significant increase in trends of the nutrient intake by quartiles of the corresponding PC (*P*<0.001). For the nutrients that were not constituent elements of the other PCs, the trends were either weakened or reversed.

**Figure 1:**
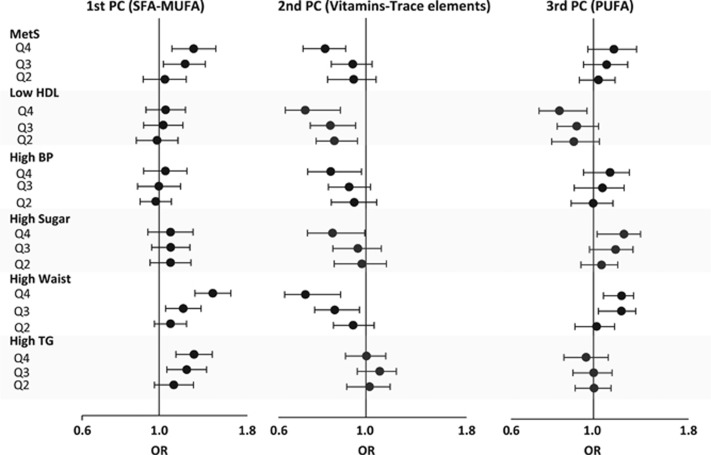
Comparing adjusted odds ratio of the quartiles of each principle component with the fourth quartile as reference to predict metabolic syndrome (MetS) and its constituents (adjusted for age, sex and race).

We used logistic regression to examine the strength of the association of MetS and its components with PCs representative of dietary patterns in fully adjusted models (Figure 1). There was a graded increase in odds of association with high waist and high triglyceride by quartiles of saturated/monounsaturated fatty acids (the first PC), so that the fourth quartile was associated with a 1.43-fold (95% confidence interval (CI): 1.27–1.61, *P*<0.001) higher odds of association with high waist and 1.26-fold (95% CI: 1.12–1.43, *P*<0.001) higher odds of association with high triglyceride when compared with the first quartile. Similarly, the highest quartile was associated with 1.27-fold (95% CI: 1.10–1.46, *P*=0.001) higher odds of association with MetS when compared with the first quartile. There was no association between the first PC and low HDL, high blood pressure, and high blood sugar criteria. On the other hand, the highest quartile for a PUFA diet was associated with lower odds of having unfavorable HDL, but higher odds of elevated blood sugar and higher waist, so that Q4 had an odds ratio of 0.81 (95% CI: 0.72–0.91, *P*<0.001) for association with low HDL, but had 1.21-fold (95% CI: 1.05–1.39, *P*=0.011) higher odds of association with elevated blood sugar, and 1.19-fold (95% CI: 1.08–1.32, *P*=0.001) higher odds of association with high waist when compared with Q1. There was no association between PUFA diet and high blood pressure, high triglycerides and presence of MetS in general. Similarly, the highest quartile of vitamins and trace elements (the second PC) was associated with significantly lower odds of unfavorable HDL, elevated blood pressure, elevated blood sugar, high waist and the presence of MetS in general, so that an odds ratio of association of Q4 with MetS was 0.79 (95% CI: 0.70–0.89, *P*<0.001) when compared with Q1. The PC representative of vitamins and trace elements was not associated with high serum triglycerides.

### Nutrient-by-nutrient approach

Using analysis of covariance, we compared the age- gender- and race-adjusted mean of nutrient intakes and ranked them by nominal *P*-value from the most significant difference by categories of MetS (Table 3) and its components (Supplementary Tables 1–5). Accordingly the top 2 nutrients associated with high blood pressure were a higher intake of alcohol but lower dietary fiber consumption; for the high glucose criterion, they were lower levels of added vitamin B12 and vitamin E; for low HDL criterion, they were lower intake of alcohol and lower magnesium intake; for high triglycerides, they were high carbohydrate and total sugar intake; and for high waist criterion, they were higher intake of hexadecenoic acid (MFA 16:1) and octadecanoic acid (SFA 18:1; *P*⩽0.0005). Similarly, Table 3 shows that the top 2 nutrients associated with the presence of MetS were lower dietary magnesium and lower dietary fiber intake (*P*<0.000001).

**Figure 2:**
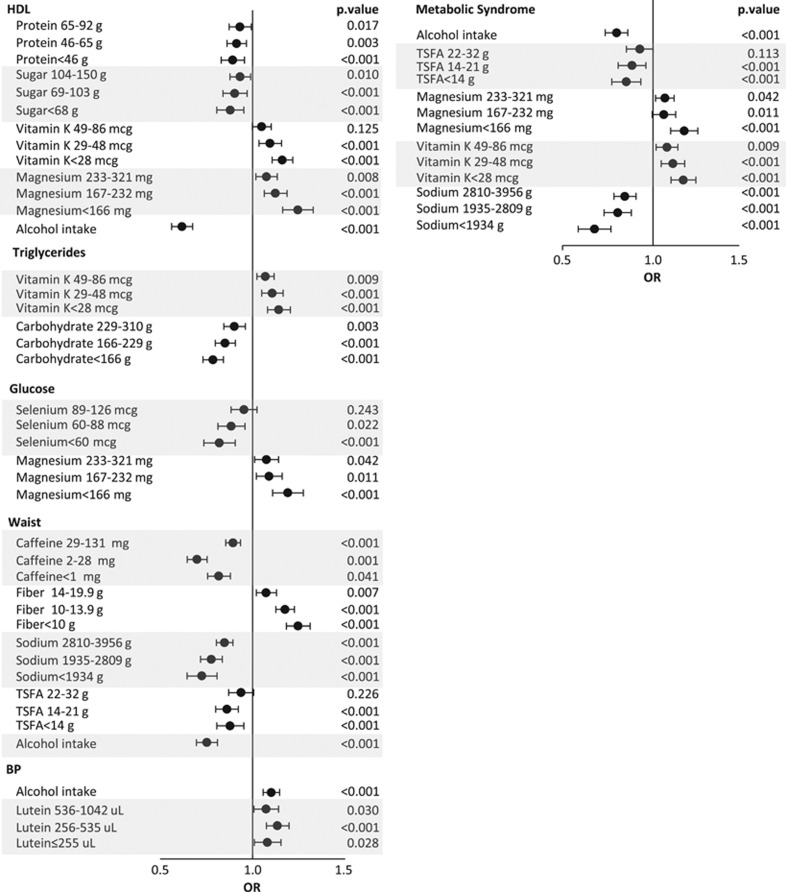
Comparing age-, sex- and race-adjusted odds ratio of the first to third quartiles of the nutrients with the fourth quartile as reference by metabolic syndrome (right panel) and its components (left panel), using multiple logistic regression analysis in a nutrient-by-nutrient approach. TSFA, total saturated fatty acids. *P-*values pertain to the comparison of each quartile to the fourth quartile except for alcohol intake which is compared with no alcohol intake.

We then applied multiple logistic regression models adjusting for age, gender, race (as covariates) and nutrients (as predictors) to identify the nutrients independently associated with each criterion as well as with MetS. To do so, each nutrient was classified into quartiles and odds ratios were calculated by comparing odds of association for each quartile with the fourth quartile as the reference. According to Figure 2 (left panel), intake of alcohol as well as lower intake of protein and sugar was associated with lower risk of the presence of HDL criterion. On the other hand, lower intake of magnesium and vitamin K was associated with higher risk of HDL criterion. For triglyceride criterion, lower carbohydrate and vitamin K consumption were associated with lower and higher risk of the presence of triglyceride criterion, respectively. For glucose criterion, lower selenium and magnesium intake were associated with lower and higher risk of elevated glucose, respectively. For waist criterion, intake of alcohol and lower consumption of caffeine, sodium and total saturated fatty acids were associated with lower risk of the presence of waist criterion. On the other hand, lower consumption of fiber was associated with higher risk of the presence of a higher waist criterion. For the blood pressure criterion, alcohol intake and lower consumption of lutein were associated with higher risk of elevated blood pressure. Similarly, Figure 2 (right panel) shows that alcohol intake and lower consumption of total saturated fatty acid and sodium were associated with lower risk of MetS, but consumption of lower magnesium and vitamin K levels was associated with higher risk of MetS.

## Discussion

In this study, we applied two complementary analytic methods including data reduction using PC analysis and a nutrient-by-nutrient approach. Using the data reduction approach, we found that the highest quartile of the first PC (representative of saturated and monounsaturated fatty acids) was associated with 1.27-fold higher odds of MetS compared with its lowest quartile. In contrast, the highest quartile of the second PC representative of vitamins and trace elements was associated with significantly lower odds of MetS, and the third PC (PUFA diet) did not have any association with MetS. On the other hand, a nutrient-by-nutrient approach showed that alcohol intake and lower consumption of total saturated fatty acid and sodium were associated with lower odds of MetS, but lower consumption of magnesium and vitamin K was associated with higher odds of MetS, an approach that disclosed significance of vitamin K and alcohol consumption in relation to MetS at population level. Similar analytic approaches carried out on components of MetS separately disclosed significant nutrients independently associated with components of MetS that were not captured by PC approach otherwise, for example, the association of sugar, vitamin K and alcohol with HDL; vitamin k with triglycerides; caffeine and alcohol with waist; and lutein with blood pressure. Specifically, lower intake of vitamin K associated with unfavorable alteration of HDL and triglycerides, and lower lutein intake associated with unfavorable alteration of blood pressure reflect novel findings requiring confirmation in further studies. The rationale for using data reduction approach together with a nutrient-by-nutrient approach is that, although the data reduction approach groups together the nutrients with high correlation coefficients and provides a global view of a large number of similar variables, it may not capture a highly significant and independent predictive nutrient that might not have been grouped together with any other PCs otherwise. As is evident from Table 2, alcohol and caffeine were not incorporated in the final PCs. In spite of that, the nutrient-by-nutrient approach showed that alcohol and caffeine were independent associated of MetS, with alcohol being the most significant associate of Mets among all other nutrients.

Association of dietary and serum SFA and PUFA with MetS was explored in several studies.^23, 24, 25, 26^ These reports are comparable with our findings of higher risk of MetS in association with SFA but not with PUFA. In this study, we also found a higher level of waist circumference and serum triglycerides at the highest quartile of the first PC. These findings are aligned with work of others who have shown increased post-prandial triglycerides with MUFA diet,^27^ upregulation of proinflammatory obesity-linked gene expression by SFA diet^25^ and SFA-mediated differential expression of ghrelin and peptide YY in favor of increased appetite,^28^ altogether suggest that SFA may promote MetS in part by alteration of triglycerides/free fatty acids cycling in favor of increased appetite and obesity. Our findings also suggest that the higher intake of the nutrients loaded in the second PC including dietary fiber, folate, vitamins-A, -Bs, -E, magnesium, iron and copper was associated with lower odds of MetS and its HDL, blood pressure, glucose, and central obesity components. These findings are in agreement with earlier studies reported salutary effects of dietary fiber,^29^ vitamin A,^30^ vitamin B12,^7, 30^ niacin,^31^ magnesium^32^ and copper^9^ on the rate of MetS. Possible mechanisms may partially be explained by improvement of insulin resistance and glucose intolerance^33^ by folate, vitamin A, -E, -B6, -B12, thiamine, magnesium and copper; improvement of endothelial function^33, 34^ by magnesium, folate, niacin and vitamin E; decrease in blood pressure^34, 35^ by niacin, thiamine, riboflavin, vitamin B12, Iron and magnesium; increased HDL and decreased oxidative stress^36, 37^ by niacin, vitamin E, magnesium and copper; and decrease in triglycerides^38^ by vitamin E, and magnesium, altogether translate to lower likelihood of MetS components and eventually lower likelihood of MetS.

The nutrient-by-nutrient approach revealed additional links with MetS and its components beyond what was observed with data reduction approach. Accordingly, we observed lower odds of MetS in association with alcohol intake, higher intake of vitamin K, but a lower intake of sodium. Possible mechanistic explanations may include vitamin K-mediated alteration of insulin sensitivity;^39^ change in renal sodium handling in favor of increased proximal renal tubular reabsorption of sodium, increasing production of glucocorticoids, worsening insulin resistance, increasing blood pressure and finally worsening obesity with higher sodium intake;^8^ increase in triglycerides and decrease in HDL^40^ with higher intake of carbohydrates; worsening glucose tolerance with higher selenium intake;^41^ and beneficial impact of lutein on cardiometabolic health.^42^ We also noted a negative correlation between caffeine consumption with central obesity. On the other hand, several meta-analyses report protective effect of caffeine in type 2 diabetes.^43, 44^ The seemingly controversial reports maybe explained by the consumption of obesogenic co-nutrients along with high caffeine consumption in general population, or by variation in rate of caffeine metabolism dictated by CYP1A2 variant leading to differential association of caffeine intake with MetS.^45^ Association of MetS with alcohol intake is also complex and depends on the dose and frequency of alcohol intake explaining both the increased^46, 47^ and decreased^48^ rate of MetS reported by different studies. Similarly, the timing of alcohol intake impacts the magnitude and the direction of change in blood pressure.^49^ The observed lower odds of MetS with alcohol intake in this study is because alcohol intake on average and at the national level is mild and therefore the detrimental effect of heavy alcohol intake on MetS is not captured in this analysis.

Several novel aspects of this study are worth to mention. There is an important need for population-based interventions to halt or slow down the rapidly increasing prevalence of MetS. A crucial aspect of effective interventions is the need for a thorough understanding of the dietary interplays of MetS as intervening targets. Dietary patterns are viewed by many researchers as a tool to study the dietary habits in a population.^13, 50, 51, 52, 53^ However, the limitations of such approach are less well recognized. In this study, we showed that exclusive reliance on the dietary patterns determined by application of PCA can miss the opportunity of detecting significant links of MetS with nutrients that are not included in the dietary patterns. For example, although the associations of carbohydrate and alcohol intake with MetS are well-known established associations, only carbohydrate intake was included in the dietary patterns and not alcohol. Therefore, the studies of dietary habits that are based exclusively on the dietary patterns may carry significant gaps. We also performed similar analysis on each one of the five criteria of MetS. By doing so, we disclosed criterion-specific links with the nutrients that have not necessarily been identical with the associates of MetS, for example, the association of protein and sugar with HDL, carbohydrate with triglycerides, selenium with glucose, caffeine with waist and lutein with blood pressure using the nutrient-by-nutrient approach. From practical standpoint, this means that we are illuminating intervening targets specific to each criterion rather than the full blown MetS, which may have the potential for establishing preventive measures at early stages. To that end, although many of the observed associations may sound confirmatory, the real value of these observations is identification of intervening targets in the nationally representative general population. Last but not least, despite the confirmatory nature of many of the associations, the lower intake of vitamin K associated with unfavorable alteration of HDL and triglycerides, and lower lutein intake associated with unfavorable alteration of blood pressure may reflect novel findings requiring confirmation in further studies.

This study has several strengths. To our knowledge, it is the largest study of the association of dietary patterns with MetS. The study is sufficiently powered to test the associations. The selection of the participants was based on random sampling of the general population and therefore the results obtained from nationally representative samples can be extrapolated to the general population. As the data collection was performed on all days of the week throughout the year in NHANES, the potential for selection bias is very low.^54, 55^ We implemented two complementary rigorous analytic approaches and have provided a comprehensive view on the dietary signature of MetS. To that end, some of the observations are likely novel that may serve as the basis for further future research, for example, the association of vitamin K with HDL and triglyceride components of MetS.

This study also has important limitations. As this is a cross-sectional observation, it does not allow inferring causality. Our nutrient-by-nutrient analytic approach revealed the correlates of MetS and its components only with the most significant independent nutrients and therefore does not provide the most inclusive list of involved nutrients. The possibility of residual confounders from unmeasured nutrients or other variables is not entirely ruled out. The impact of the other determinants of MetS such educational level, income inequality and physical activity is beyond the scope of this study and requires separate in-depth analyses in future research. Interpretation of our findings should be with caution; several trials have shown increase of glucose and higher incidence of diabetes with pharmacologic use of niacin.^56, 57, 58^ Lower odds of having high glucose at higher categories of physiologic niacin intake in our study is likely reflection of lower amounts in diet, but alternate dietary nutrients offsetting this negative effect can also be a possibility. Metabolic derangements in association with heavy alcohol intake are also a well-known phenomenon. Therefore, lower odds of MetS with alcohol intake should not simply be interpreted as its preventive effect on MetS, rather the association is the reflection of mild alcohol intake (12.7 g on average in individuals with Mets: Supplementary Table 2) at the national level. The use of 1 day of 24-h dietary recall may not fully capture the long-term dietary behaviors as well as the seasonal variation of the dietary habits. However, these concerns are mitigated by the large sample size and continuous random sampling throughout the year increasing the probability of inclusion of diverse dietary behaviors.

This study has important clinical and public health implications. Comprehensive understanding about the dietary interplays of MetS and its components is a necessary and important step towards public health policy making and raising public awareness. Our study provides a comprehensive snapshot of dietary correlates of MetS at the national level. Further analyses by components of MetS provide additional insight about pathways of progression to MetS amenable to dietary interventions. Although our findings reinforce the importance of balanced diet, the disclosed links between some of the nutrients and metabolic derangements may represent novel metabolic pathways and basis for further and future research.

In conclusion, our findings provide further evidence on the association between high intakes of SFA-enriched diet with higher risk of MetS, whereas a nutrient pattern characterized by high consumption of vitamins was associated with lower odds of MetS. Provision of additional independent links with MetS and its components, beyond what was presented by PCA-derived nutrient patterns suggests that the studies of dietary habits in populations require complementary analytic approaches, and that the studies that exclusively rely on the PCA-derived dietary patterns may not fully represent the dietary habits in general population as well as their links with the clinical phenotypes. Identification of independent associates of MetS and its components proposed intervening targets to halt progression of MetS. Further studies are required to assess efficacy of dietary intervention on proposed targets on incident rate of MetS.

## Figures and Tables

**Table 1:** Distribution of demographics and components of metabolic syndrome by status of metabolic syndrome

*Variables*	*With MetS*	*Without MetS*
Unweighted *N*	6561	16 596
Weighted estimates per cycle-year	20 205 996	54 270 122
Age (mean (95% CI))*	52.6 (51.9–53.3)	43.5 (42.8–44.2)
		
*Gender (%)*		
Male	49.4	47.9
Female	50.6	52.1
		
Race (%)*		
Non-Hispanic White	71.7	67.3
Mexican American	9.1	8.0
Non-Hispanic Black	8.8	12.6
Other Hispanic	5.0	5.1
Others	5.4	7.0
Glucose criterion*	61.6	15.7
Triglyceride criterion*	63.0	9.0
HDL criterion*	63.5	13.6
Blood pressure criterion*	53.0	17.3
Waist criterion*	47.6	5.8

Abbreviations: CI, confidence interval; HDL, high-density lipid; MetS, metabolic syndrome.

*P<0.001.

**Table 2:**
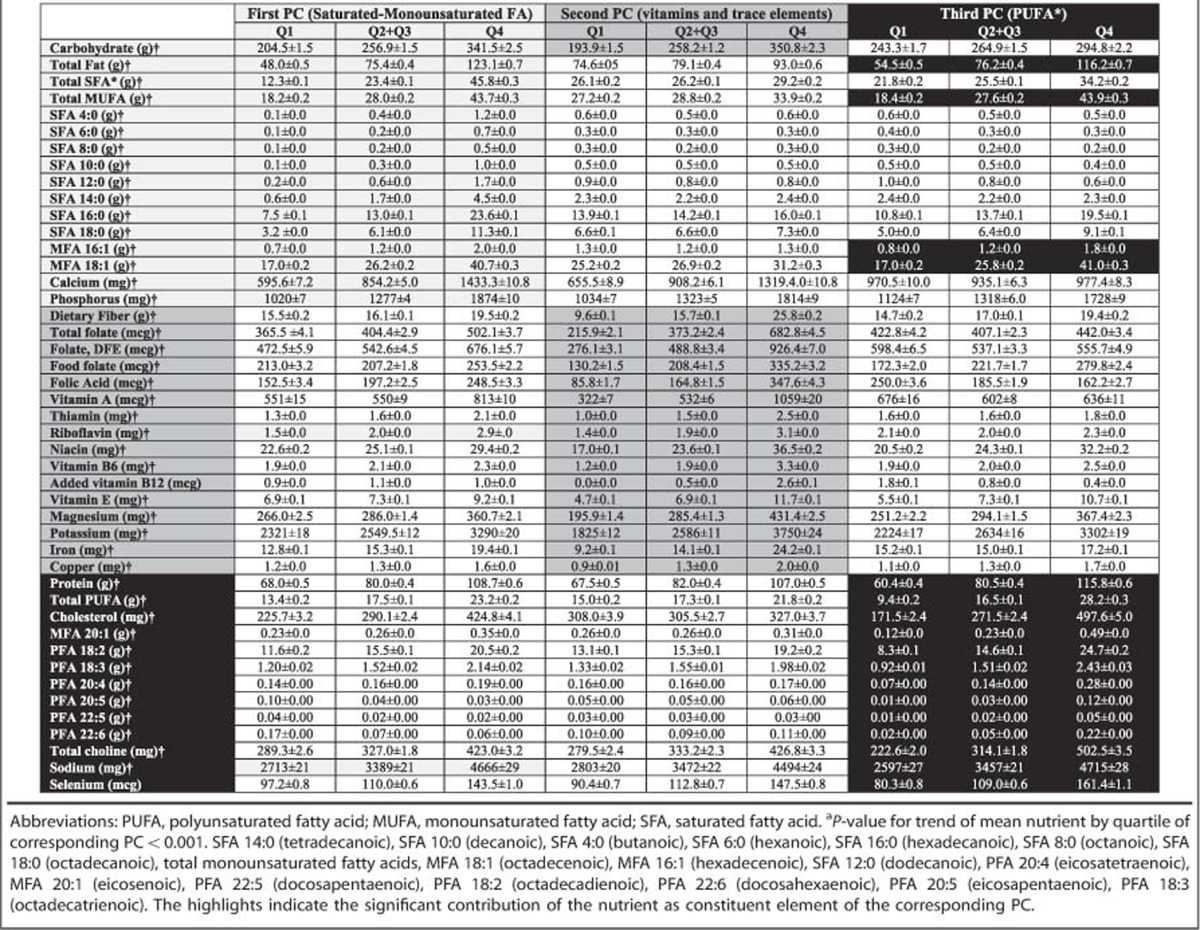
Age-, gender- and race-adjusted mean of nutrient intakes across quartiles of principle component scores representative of nutrient patterns

**Table 3:** Comparing adjusted mean of dietary components by presence of at least three components of metabolic syndrome (adjusted for age, sex and race)

*Variables*	*Metabolic syndrome criteria*		
	*Absence*	*Presence*	P-*value*
Magnesium (mg)	299.81	283.49	**2.5E−08**
Dietary fiber (g)	16.979	15.89	**6.7E−07**
MFA 16:1 (hexadecenoic; g)	1.1757	1.2562	**1.5E−05**
Vitamin C (mg)	94.1773	86.6496	**2.6E−05**
Added vitamin B12 (μg)	0.887	0.6913	**2.7E−05**
Vitamin E as alpha-tocopherol (mg)	7.609	7.0532	**2.8E−05**
Lutein+zeaxanthin (μg)	1534.76	1263.63	**5.8E−05**
SFA 18:0 (octadecanoic) (g)	6.2792	6.7221	**1.4E−04**
Vitamin B6 (mg)	2.051	1.957	**1.4E−04**
Food folate (μg)	222.55	210.21	**1.5E−04**
Beta-carotene (μg)	2221.58	1864.08	**1.0E−03**
Sodium (mg)	3452.36	3593.83	**1.0E−03**
Vitamin K (μg)	108.1161	91.4343	**2.0E−03**
Alcohol (g)	10.3378	8.2825	**2.0E−03**
PFA 22:6 (docosahexaenoic; g)	0.0899	0.0767	**2.0E−03**
SFA 16:0 (hexadecanoic; g)	13.5096	14.1984	**3.0E−03**
Total saturated fatty acids (g)	24.6555	25.9104	**4.0E−03**
Cholesterol (mg)	292.7	307.05	**7.0E−03**
Potassium (mg)	2663.96	2591.77	**8.0E−03**
PFA 20:4 (eicosatetraenoic; g)	0.1539	0.1613	**9.0E−03**
Riboflavin (vitamin B2; mg)	2.0337	1.9717	1.3E−02
Copper (mg)	1.3311	1.2708	1.9E−02
PFA 20:5 (eicosapentaenoic; g)	0.0474	0.0399	2.0E−02
Total monounsaturated fatty acids (g)	28.2386	29.2891	2.3E−02
Vitamin A, RAE (μg)	595.16	546.57	2.3E−02
Added alpha-tocopherol (mg)	0.447	0.3095	2.9E−02
Total fat (g)	77.084	79.7601	3.0E−02
MFA 18:1 (octadecenoic; g)	26.372	27.3251	3.0E−02
Alpha-carotene (μg)	430.87	367.29	4.1E−02
Total folate (μg)	407.46	397.35	4.3E−02
Niacin (mg)	25.1137	24.6133	4.6E−02
Zinc (mg)	11.5234	11.9959	6.4E−02
Beta-cryptoxanthin (μg)	113.52	104.96	7.0E−02
SFA 14:0 (tetradecanoic; g)	1.9903	2.0668	7.2E−02
Energy (kcal)	2112.55	2141.79	1.5E−01
Total sugars (g)	113.9629	116.2604	1.8E−01
Protein (g)	82.2884	83.4037	2.0E−01
Carbohydrate (g)	260.6908	263.6338	2.1E−01
Folate, DFE (μg)	537.11	528.51	2.2E−01
Calcium (mg)	906.55	890.76	2.4E−01
Retinol (μg)	387.44	371.61	2.5E−01
Thiamine (vitamin B1; mg)	1.6081	1.5913	3.2E−01
PFA 18:3 (octadecatrienoic; g)	1.5713	1.5477	3.9E−01
Vitamin B12 (μg)	5.1096	5.0144	4.8E−01
PFA 22:5 (docosapentaenoic; g)	0.0247	0.0241	4.8E−01
MFA 20:1 (eicosenoic; g)	0.2637	0.2677	4.9E−01
Folic acid (μg)	184.95	187.14	5.3E−01
Caffeine (mg)	135.26	138.05	5.6E−01
PFA 18:2 (octadecadienoic; g)	15.431	15.5826	5.9E−01
Selenium (μg)	113.1357	113.879	5.9E−01
MFA 22:1 (docosenoic; g)	0.0324	0.0309	6.0E−01
SFA 12:0 (dodecanoic; g)	0.6881	0.6997	6.1E−01
Iron (mg)	15.1119	15.0318	6.3E−01
Total polyunsaturated fatty acids (g)	17.5277	17.6427	7.1E−01
Total choline (mg)	333.7357	335.2332	7.3E−01
PFA 18:4 (octadecatetraenoic; g)	0.01424	0.01396	7.7E−01
Theobromine (mg)	32.37	31.88	7.8E−01
SFA 8:0 (octanoic; g)	0.2229	0.222	8.8E−01
SFA 10:0 (decanoic; g)	0.4054	0.4068	8.9E−01
Lycopene (μg)	5056.35	5037.6	9.1E−01
Phosphorus (mg)	1327.95	1326.29	9.1E−01
SFA 4:0 (butanoic; g)	0.487	0.4881	9.3E−01
SFA 6:0 (hexanoic; g)	0.27	0.2696	9.5E−01

Abbreviations: MFA, monounsaturated fatty acids; PFA, polyunsaturated fatty acid; SFA, saturated fatty acids. The bold *P-*values have passed the Benjamini–Hochberg False Discovery Threshold at *P*<0.05.
