# Supplementary material for: Link of dietary patterns with metabolic syndrome: analysis of the National Health and Nutrition Examination Survey
Source: Nutr Diabetes. 2017 Mar 20;7(3):e255–. doi: 10.1038/nutd.2017.11 (PMC5380894; doi:10.1038/nutd.2017.11)
Supplement: Supplementary Information [file nutd201711x1.docx]

**Supplementary data:**

**Title: Link of Dietary Patterns with Metabolic Syndrome: Analysis of the National Health and Nutrition Examination Survey**

**Authors:** Mohsen Mazidi^1,2^ , Subramaniam Pennathur^3^, Farsad Afshinnia^3^

^1^ Key State Laboratory of Molecular Developmental Biology, Institute of Genetics and Developmental Biology, Chinese Academy of Sciences, Beijing 100101, China

^2^ Institute of Genetics and Developmental Biology, International College, University of Chinese Academy of Science, Beijing 100101, China

^3^ Division of Nephrology, Department of Internal Medicine, University of Michigan, Ann Arbor, Michigan

**Running title:** Dietary Pattern and Metabolic Syndrome

**Journal:** Nutrition and Diabetes

**Variables used from NHANES publically available datasets in this study:**

| **Name** | **Label** |
| --- | --- |
| RIAGENDR | Gender |
| RIDAGEYR | Age in years at screening |
| RIDRETH1 | Race/Hispanic origin |
| SDMVPSU | Masked variance pseudo-PSU |
| SDMVSTRA | Masked variance pseudo-stratum |
| DR1TPROT | Protein (gm) |
| DR1TCARB | Carbohydrate (gm) |
| DR1TSUGR | Total sugars (gm) |
| DR1TFIBE | Dietary fiber (gm) |
| DR1TTFAT | Total fat (gm) |
| DR1TSFAT | Total saturated fatty acids (gm) |
| DR1TMFAT | Total monounsaturated fatty acids (gm) |
| DR1TPFAT | Total polyunsaturated fatty acids (gm) |
| DR1TCHOL | Cholesterol (mg) |
| DR1TATOC | Vitamin E as alpha-tocopherol (mg) |
| DR1TATOA | Added alpha-tocopherol (Vitamin E) (mg) |
| DR1TRET | Retinol (mcg) |
| DR1TVARA | Vitamin A, RAE (mcg) |
| DR1TACAR | Alpha-carotene (mcg) |
| DR1TBCAR | Beta-carotene (mcg) |
| DR1TCRYP | Beta-cryptoxanthin (mcg) |
| DR1TLYCO | Lycopene (mcg) |
| DR1TLZ | Lutein + zeaxanthin (mcg) |
| DR1TVB1 | Thiamin (Vitamin B1) (mg) |
| DR1TVB2 | Riboflavin (Vitamin B2) (mg) |
| DR1TNIAC | Niacin (mg) |
| DR1TVB6 | Vitamin B6 (mg) |
| DR1TFOLA | Total folate (mcg) |
| DR1TFA | Folic acid (mcg) |
| DR1TFF | Food folate (mcg) |
| DR1TFDFE | Folate, DFE (mcg) |
| DR1TCHL | Total choline (mg) |
| DR1TVB12 | Vitamin B12 (mcg) |
| DR1TB12A | Added vitamin B12 (mcg) |
| DR1TVC | Vitamin C (mg) |
| DR1TVK | Vitamin K (mcg) |
| DR1TCALC | Calcium (mg) |
| DR1TPHOS | Phosphorus (mg) |
| DR1TMAGN | Magnesium (mg) |
| DR1TIRON | Iron (mg) |
| DR1TZINC | Zinc (mg) |
| DR1TCOPP | Copper (mg) |
| DR1TSODI | Sodium (mg) |
| DR1TPOTA | Potassium (mg) |
| DR1TSELE | Selenium (mcg) |
| DR1TCAFF | Caffeine (mg) |
| DR1TTHEO | Theobromine (mg) |
| DR1TALCO | Alcohol (gm) |
| DR1TMOIS | Moisture (gm) |
| DR1TS040 | SFA 4:0 (Butanoic) (gm) |
| DR1TS060 | SFA 6:0 (Hexanoic) (gm) |
| DR1TS080 | SFA 8:0 (Octanoic) (gm) |
| DR1TS100 | SFA 10:0 (Decanoic) (gm) |
| DR1TS120 | SFA 12:0 (Dodecanoic) (gm) |
| DR1TS140 | SFA 14:0 (Tetradecanoic) (gm) |
| DR1TS160 | SFA 16:0 (Hexadecanoic) (gm) |
| DR1TS180 | SFA 18:0 (Octadecanoic) (gm) |
| DR1TM161 | MFA 16:1 (Hexadecenoic) (gm) |
| DR1TM181 | MFA 18:1 (Octadecenoic) (gm) |
| DR1TM201 | MFA 20:1 (Eicosenoic) (gm) |
| DR1TM221 | MFA 22:1 (Docosenoic) (gm) |
| DR1TP182 | PFA 18:2 (Octadecadienoic) (gm) |
| DR1TP183 | PFA 18:3 (Octadecatrienoic) (gm) |
| DR1TP184 | PFA 18:4 (Octadecatetraenoic) (gm) |
| DR1TP204 | PFA 20:4 (Eicosatetraenoic) (gm) |
| DR1TP205 | PFA 20:5 (Eicosapentaenoic) (gm) |
| DR1TP225 | PFA 22:5 (Docosapentaenoic) (gm) |
| DR1TP226 | PFA 22:6 (Docosahexaenoic) (gm) |
| BMXWT | Weight (kg) |
| BMXHT | Standing Height (cm) |
| BMXBMI | Body Mass Index (kg/m^2^) |
| BMXWAIST | Waist Circumference (cm) |
| LBXSGL | Glucose, serum (mg/dL) |
| LBXSTR | Triglycerides (mg/dL) |
| LBDHDD | Direct HDL-Cholesterol (mg/dL) |
| BPXSY1 | Systolic: Blood pres (1st rdg) mm Hg |
| BPXSY2 | Systolic: Blood pres (2nd rdg) mm Hg |
| BPXSY3 | Systolic: Blood pres (3rd rdg) mm Hg |
| BPXSY4 | Systolic: Blood pres (4th rdg) mm Hg |
| BPXDI1 | Diastolic: Blood pres (1st rdg) mm Hg |
| BPXDI2 | Diastolic: Blood pres (2nd rdg) mm Hg |
| BPXDI3 | Diastolic: Blood pres (3rdt rdg) mm Hg |
| BPXDI4 | Diastolic: Blood pres (4th rdg) mm Hg |

**Table 1:** Comparing age, sex, and race adjusted mean of dietary components by presence of blood pressure criteria

| **Variables** | **Blood Pressure criteria** | | |
| --- | --- | --- | --- |
|  | **Absence** | **Presence** | **P value** |
| **Alcohol (gm)** | 8.4551 | 12.7541 | 4.0E-07 |
| **Dietary fiber (gm)** | 16.9969 | 15.8802 | 6.0E-06 |
| **Vitamin A, RAE (mcg)** | 605.82 | 526.05 | 1.1E-04 |
| **Beta-carotene (mcg)** | 2246.11 | 1815.68 | 1.3E-04 |
| **Riboflavin (Vitamin B2) (mg)** | 2.048 | 1.9444 | 2.7E-04 |
| **Vitamin E as alpha-tocopherol (mg)** | 7.5981 | 7.1409 | 1.0E-03 |
| **Lutein + zeaxanthin (mcg)** | 1524.73 | 1285.12 | 1.0E-03 |
| **Copper (mg)** | 1.3359 | 1.2604 | 1.0E-03 |
| **Potassium (mg)** | 2670.25 | 2579.9 | 1.0E-03 |
| **Caffeine (mg)** | 141.88 | 125.54 | 1.0E-03 |
| **Retinol (mcg)** | 395.84 | 355.92 | 2.0E-03 |
| **MFA 22:1 (Docosenoic) (gm)** | 0.0296 | 0.0375 | 2.0E-03 |
| **Food folate (mcg)** | 220.97 | 213.77 | 3.0E-03 |
| **Magnesium (mg)** | 298.43 | 287.66 | 3.0E-03 |
| **Alpha-carotene (mcg)** | 440.55 | 346.64 | 6.0E-03 |
| **Theobromine (mg)** | 34.01 | 29.03 | 6.0E-03 |
| **Added vitamin B12 (mcg)** | 0.8737 | 0.7319 | 9.0E-03 |
| **Vitamin C (mg)** | 92.9404 | 88.6224 | 9.0E-03 |
| **Vitamin K (mcg)** | 108.3616 | 91.7909 | 1.3E-02 |
| **Iron (mg)** | 15.2138 | 14.769 | 2.0E-02 |
| **Total folate (mcg)** | 407.46 | 395.65 | 2.1E-02 |
| **Vitamin B6 (mg)** | 2.0413 | 1.9781 | 3.8E-02 |
| **Thiamin (Vitamin B1) (mg)** | 1.6114 | 1.5752 | 4.6E-02 |
| **Folate, DFE (mcg)** | 538.19 | 523.17 | 5.2E-02 |
| **Vitamin B12 (mcg)** | 5.1765 | 4.8707 | 5.8E-02 |
| **Calcium (mg)** | 909.33 | 885.71 | 7.2E-02 |
| **PFA 18:3 (Octadecatrienoic) (gm)** | 1.5811 | 1.5329 | 7.4E-02 |
| **Lycopene (mcg)** | 4973.16 | 5282.42 | 9.0E-02 |
| **Total sugars (gm)** | 115.44 | 112.4968 | 9.4E-02 |
| **SFA 8:0 (Octanoic) (gm)** | 0.2255 | 0.2161 | 1.2E-01 |
| **MFA 16:1 (Hexadecenoic) (gm)** | 1.1881 | 1.2194 | 1.2E-01 |
| **Phosphorus (mg)** | 1332.84 | 1313.42 | 1.5E-01 |
| **MFA 20:1 (Eicosenoic) (gm)** | 0.2619 | 0.2715 | 1.5E-01 |
| **Added alpha-tocopherol (Vitamin E) (mg)** | 0.4281 | 0.3468 | 1.9E-01 |
| **SFA 6:0 (Hexanoic) (gm)** | 0.2726 | 0.2633 | 2.2E-01 |
| **Carbohydrate (gm)** | 262.1037 | 258.7334 | 2.4E-01 |
| **SFA 10:0 (Decanoic) (gm)** | 0.4097 | 0.3976 | 2.4E-01 |
| **Folic acid (mcg)** | 186.52 | 181.9 | 2.5E-01 |
| **PFA 22:5 (Docosapentaenoic) (gm)** | 0.0242 | 0.0252 | 3.1E-01 |
| **Total polyunsaturated fatty acids (gm)** | 17.6653 | 17.4002 | 3.7E-01 |
| **PFA 18:2 (Octadecadienoic) (gm)** | 15.567 | 15.3377 | 3.9E-01 |
| **Zinc (mg)** | 11.6807 | 11.4959 | 4.1E-01 |
| **Total choline (mg)** | 333.1119 | 336.4514 | 4.3E-01 |
| **SFA 18:0 (Octadecanoic) (gm)** | 6.3816 | 6.4685 | 4.3E-01 |
| **SFA 4:0 (Butanoic) (gm)** | 0.4905 | 0.4798 | 4.5E-01 |
| **PFA 20:4 (Eicosatetraenoic) (gm)** | 0.1556 | 0.158 | 4.5E-01 |
| **Cholesterol (mg)** | 295.84 | 300.15 | 4.6E-01 |
| **PFA 20:5 (Eicosapentaenoic) (gm)** | 0.0441 | 0.0464 | 4.7E-01 |
| **Sodium (mg)** | 3478.82 | 3498.25 | 5.1E-01 |
| **Beta-cryptoxanthin (mcg)** | 109.42 | 106.89 | 5.7E-01 |
| **SFA 12:0 (Dodecanoic) (gm)** | 0.6975 | 0.6825 | 6.1E-01 |
| **SFA 16:0 (Hexadecanoic) (gm)** | 13.6776 | 13.7761 | 6.4E-01 |
| **Niacin (mg)** | 24.9771 | 24.868 | 6.8E-01 |
| **Total saturated fatty acids (gm)** | 24.9725 | 25.1319 | 6.9E-01 |
| **PFA 18:4 (Octadecatetraenoic) (gm)** | 0.01397 | 0.01435 | 7.0E-01 |
| **PFA 22:6 (Docosahexaenoic) (gm)** | 0.0852 | 0.0868 | 7.1E-01 |
| **Total monounsaturated fatty acids (gm)** | 28.5203 | 28.6307 | 8.2E-01 |
| **Selenium (mcg)** | 113.1094 | 113.3806 | 8.3E-01 |
| **MFA 18:1 (Octadecenoic) (gm)** | 26.6414 | 26.7028 | 8.9E-01 |
| **SFA 14:0 (Tetradecanoic) (gm)** | 2.0109 | 2.014 | 9.4E-01 |
| **Protein (gm)** | 82.5344 | 82.5575 | 9.8E-01 |
| **Total fat (gm)** | 77.8935 | 77.9005 | 1.0E+00 |

MFA: Mono unsaturated Fatty Acids; SFA: Saturated fatty acids; PFA: Poly unsaturated fatty acid. The bold p values have passed the Benjamini–Hochberg False Discovery Threshold at p<0.05.

**Table 2:** Comparing age, sex, and race adjusted mean of dietary components by presence of glucose criterion

| **Variables** | **Glucose criterion** | | |
| --- | --- | --- | --- |
|  | **Absence** | **Presence** | **P value** |
| **Added vitamin B12 (mcg)** | 0.9102 | 0.7001 | 3.2E-05 |
| **Added alpha-tocopherol (Vitamin E) (mg)** | 0.467 | 0.3111 | 4.5E-04 |
| **Vitamin E as alpha-tocopherol (mg)** | 7.6102 | 7.2121 | 2.0E-03 |
| **Magnesium (mg)** | 299.04 | 288.44 | 3.0E-03 |
| **PFA 20:4 (Eicosatetraenoic) (gm)** | 0.1531 | 0.162 | 6.0E-03 |
| **Cholesterol (mg)** | 292.12 | 306.36 | 8.0E-03 |
| **MFA 16:1 (Hexadecenoic) (gm)** | 1.1839 | 1.2351 | 1.1E-02 |
| **Total sugars (gm)** | 116.1962 | 111.5467 | 1.7E-02 |
| **Sodium (mg)** | 3460.95 | 3546.1 | 1.7E-02 |
| **Niacin (mg)** | 25.1952 | 24.585 | 2.1E-02 |
| **Riboflavin (Vitamin B2) (mg)** | 2.0419 | 1.9844 | 2.8E-02 |
| **Vitamin B6 (mg)** | 2.0463 | 1.9878 | 3.1E-02 |
| **Food folate (mcg)** | 221.39 | 215.71 | 3.6E-02 |
| **Carbohydrate (gm)** | 263.4632 | 258.2637 | 5.5E-02 |
| **Dietary fiber (gm)** | 16.8842 | 16.4199 | 6.1E-02 |
| **Total folate (mcg)** | 408.4 | 400.02 | 7.4E-02 |
| **Vitamin C (mg)** | 93.5802 | 89.7138 | 9.7E-02 |
| **SFA 18:0 (Octadecanoic) (gm)** | 6.3727 | 6.5252 | 1.2E-01 |
| **Copper (mg)** | 1.3297 | 1.2872 | 1.2E-01 |
| **Vitamin A, RAE (mcg)** | 589.37 | 569.5 | 1.3E-01 |
| **Thiamin (Vitamin B1) (mg)** | 1.6154 | 1.5869 | 1.4E-01 |
| **Potassium (mg)** | 2659.38 | 2623.15 | 1.4E-01 |
| **Retinol (mcg)** | 391.7 | 369.36 | 1.5E-01 |
| **Folate, DFE (mcg)** | 539.5 | 529.24 | 1.5E-01 |
| **Alcohol (gm)** | 10.0017 | 9.0111 | 1.6E-01 |
| **Iron (mg)** | 15.2027 | 14.9754 | 1.7E-01 |
| **SFA 16:0 (Hexadecanoic) (gm)** | 13.6519 | 13.91 | 2.1E-01 |
| **Calcium (mg)** | 909.9 | 892.39 | 2.2E-01 |
| **Beta-cryptoxanthin (mcg)** | 113.16 | 108.04 | 2.2E-01 |
| **Total monounsaturated fatty acids (gm)** | 28.4558 | 28.9462 | 2.3E-01 |
| **Theobromine (mg)** | 32.81 | 30.81 | 2.5E-01 |
| **Total choline (mg)** | 332.4209 | 336.8862 | 2.8E-01 |
| **MFA 18:1 (Octadecenoic) (gm)** | 26.582 | 26.9979 | 2.8E-01 |
| **Total saturated fatty acids (gm)** | 24.9388 | 25.3533 | 2.9E-01 |
| **Total fat (gm)** | 77.6807 | 78.7054 | 3.4E-01 |
| **PFA 22:5 (Docosapentaenoic) (gm)** | 0.0242 | 0.0251 | 3.6E-01 |
| **Selenium (mcg)** | 112.9923 | 113.9573 | 4.0E-01 |
| **MFA 20:1 (Eicosenoic) (gm)** | 0.2639 | 0.2678 | 4.7E-01 |
| **Vitamin B12 (mcg)** | 5.1222 | 5.0177 | 4.9E-01 |
| **Zinc (mg)** | 11.6284 | 11.7682 | 4.9E-01 |
| **Folic acid (mcg)** | 187.03 | 184.34 | 4.9E-01 |
| **SFA 4:0 (Butanoic) (gm)** | 0.4927 | 0.4844 | 5.2E-01 |
| **Lycopene (mcg)** | 5071.53 | 5200.02 | 5.8E-01 |
| **Phosphorus (mg)** | 1331.14 | 1323 | 6.0E-01 |
| **Protein (gm)** | 82.5289 | 82.9268 | 6.1E-01 |
| **SFA 8:0 (Octanoic) (gm)** | 0.2237 | 0.2209 | 6.2E-01 |
| **SFA 14:0 (Tetradecanoic) (gm)** | 2.0115 | 2.0313 | 6.5E-01 |
| **PFA 18:3 (Octadecatrienoic) (gm)** | 1.5707 | 1.5612 | 7.0E-01 |
| **Alpha-carotene (mcg)** | 406.32 | 419.29 | 7.0E-01 |
| **SFA 6:0 (Hexanoic) (gm)** | 0.272 | 0.2693 | 7.1E-01 |
| **Caffeine (mg)** | 138.38 | 136.73 | 7.2E-01 |
| **MFA 22:1 (Docosenoic) (gm)** | 0.0314 | 0.0324 | 7.6E-01 |
| **PFA 20:5 (Eicosapentaenoic) (gm)** | 0.0451 | 0.0444 | 7.8E-01 |
| **Vitamin K (mcg)** | 102.9151 | 105.3608 | 7.8E-01 |
| **SFA 10:0 (Decanoic) (gm)** | 0.4081 | 0.4056 | 8.0E-01 |
| **PFA 22:6 (Docosahexaenoic) (gm)** | 0.0858 | 0.0849 | 8.4E-01 |
| **PFA 18:4 (Octadecatetraenoic) (gm)** | 0.01415 | 0.01398 | 8.4E-01 |
| **Beta-carotene (mcg)** | 2113.41 | 2138.86 | 8.4E-01 |
| **PFA 18:2 (Octadecadienoic) (gm)** | 15.4853 | 15.501 | 9.5E-01 |
| **Total polyunsaturated fatty acids (gm)** | 17.5732 | 17.5897 | 9.5E-01 |
| **Lutein + zeaxanthin (mcg)** | 1464.38 | 1459.03 | 9.6E-01 |
| **SFA 12:0 (Dodecanoic) (gm)** | 0.69 | 0.6913 | 9.6E-01 |

MFA: Mono unsaturated Fatty Acids; SFA: Saturated fatty acids; PFA: Poly unsaturated fatty acid. The bold p values have passed the Benjamini–Hochberg False Discovery Threshold at p<0.05.

**Table 3:** Comparing age, sex, and race adjusted mean of dietary components by presence of HDL criteria

| **Variables** | **HDL criteria** | | |
| --- | --- | --- | --- |
|  | **Absence** | **Presence** | **P value** |
| **Alcohol (gm)** | 11.815 | 5.0099 | 2.0E-17 |
| **Magnesium (mg)** | 304.3 | 276.61 | 2.0E-15 |
| **Food folate (mcg)** | 226.69 | 203.72 | 2.0E-13 |
| **Vitamin E as alpha-tocopherol (mg)** | 7.7317 | 6.9381 | 2.0E-11 |
| **Potassium (mg)** | 2698.95 | 2533.74 | 4.0E-09 |
| **Niacin (mg)** | 25.4724 | 23.9437 | 1.0E-08 |
| **Vitamin B6 (mg)** | 2.0744 | 1.9235 | 2.0E-08 |
| **Vitamin C (mg)** | 95.4721 | 85.4625 | 2.0E-08 |
| **Lutein + zeaxanthin (mcg)** | 1564.29 | 1238.82 | 6.0E-08 |
| **Dietary fiber (gm)** | 17.1086 | 15.9072 | 9.0E-08 |
| **Vitamin K (mcg)** | 110.0734 | 89.5447 | 4.0E-07 |
| **Total folate (mcg)** | 412.96 | 389.39 | 7.0E-07 |
| **MFA 22:1 (Docosenoic) (gm)** | 0.0342 | 0.0261 | 2.0E-06 |
| **Total sugars (gm)** | 111.7867 | 120.9894 | 4.0E-06 |
| **Lycopene (mcg)** | 5348.39 | 4568.08 | 4.0E-06 |
| **PFA 22:6 (Docosahexaenoic) (gm)** | 0.0907 | 0.0739 | 8.0E-06 |
| **Total choline (mg)** | 339.4697 | 321.1104 | 2.5E-05 |
| **PFA 20:5 (Eicosapentaenoic) (gm)** | 0.0483 | 0.0372 | 7.2E-05 |
| **PFA 22:5 (Docosapentaenoic) (gm)** | 0.0253 | 0.0226 | 9.4E-05 |
| **Beta-carotene (mcg)** | 2241.12 | 1855.25 | 1.2E-04 |
| **MFA 20:1 (Eicosenoic) (gm)** | 0.2709 | 0.2523 | 1.5E-04 |
| **Total polyunsaturated fatty acids (gm)** | 17.8939 | 16.9045 | 1.8E-04 |
| **Beta-cryptoxanthin (mcg)** | 115.95 | 101.58 | 2.2E-04 |
| **PFA 18:2 (Octadecadienoic) (gm)** | 15.7645 | 14.9058 | 2.6E-04 |
| **Phosphorus (mg)** | 1343.21 | 1294.89 | 4.3E-04 |
| **Riboflavin (Vitamin B2) (mg)** | 2.0541 | 1.9548 | 1.0E-03 |
| **Folate, DFE (mcg)** | 543.55 | 519.53 | 1.0E-03 |
| **Selenium (mcg)** | 114.5057 | 110.4511 | 1.0E-03 |
| **PFA 18:3 (Octadecatrienoic) (gm)** | 1.5977 | 1.503 | 1.0E-03 |
| **Protein (gm)** | 83.41 | 80.9079 | 2.0E-03 |
| **Alpha-carotene (mcg)** | 434.16 | 358.55 | 2.0E-03 |
| **Thiamin (Vitamin B1) (mg)** | 1.6229 | 1.5673 | 3.0E-03 |
| **Added vitamin B12 (mcg)** | 0.886 | 0.7477 | 3.0E-03 |
| **MFA 18:1 (Octadecenoic) (gm)** | 27.0333 | 26.0039 | 5.0E-03 |
| **Total monounsaturated fatty acids (gm)** | 28.9463 | 27.8667 | 6.0E-03 |
| **Iron (mg)** | 15.2499 | 14.8551 | 7.0E-03 |
| **Total fat (gm)** | 78.8277 | 76.1752 | 8.0E-03 |
| **Calcium (mg)** | 914.77 | 879.82 | 8.0E-03 |
| **SFA 4:0 (Butanoic) (gm)** | 0.4988 | 0.4693 | 8.0E-03 |
| **Copper (mg)** | 1.3408 | 1.2604 | 1.2E-02 |
| **SFA 10:0 (Decanoic) (gm)** | 0.4126 | 0.3943 | 1.9E-02 |
| **Vitamin A, RAE (mcg)** | 593.25 | 559.85 | 2.2E-02 |
| **SFA 6:0 (Hexanoic) (gm)** | 0.2748 | 0.2622 | 3.4E-02 |
| **Carbohydrate (gm)** | 260.3587 | 264.7158 | 6.1E-02 |
| **SFA 8:0 (Octanoic) (gm)** | 0.225 | 0.2174 | 8.5E-02 |
| **SFA 14:0 (Tetradecanoic) (gm)** | 2.0317 | 1.9813 | 1.3E-01 |
| **Caffeine (mg)** | 135.53 | 142.65 | 1.3E-01 |
| **SFA 16:0 (Hexadecanoic) (gm)** | 13.8128 | 13.5494 | 1.6E-01 |
| **Total saturated fatty acids (gm)** | 25.2092 | 24.7349 | 1.6E-01 |
| **PFA 18:4 (Octadecatetraenoic) (gm)** | 0.01443 | 0.01333 | 2.7E-01 |
| **PFA 20:4 (Eicosatetraenoic) (gm)** | 0.1568 | 0.1539 | 2.8E-01 |
| **Sodium (mg)** | 3498.11 | 3461.81 | 3.1E-01 |
| **Cholesterol (mg)** | 298.12 | 292.94 | 3.3E-01 |
| **MFA 16:1 (Hexadecenoic) (gm)** | 1.2042 | 1.191 | 4.2E-01 |
| **Vitamin B12 (mcg)** | 5.126 | 4.9925 | 4.7E-01 |
| **SFA 12:0 (Dodecanoic) (gm)** | 0.693 | 0.683 | 6.3E-01 |
| **Theobromine (mg)** | 31.93 | 32.79 | 6.3E-01 |
| **Added alpha-tocopherol (Vitamin E) (mg)** | 0.4258 | 0.3978 | 6.3E-01 |
| **SFA 18:0 (Octadecanoic) (gm)** | 6.4314 | 6.3905 | 6.6E-01 |
| **Zinc (mg)** | 11.6958 | 11.5967 | 6.8E-01 |
| **Folic acid (mcg)** | 186.3 | 185.69 | 8.7E-01 |
| **Retinol (mcg)** | 383.67 | 386.13 | 8.7E-01 |

MFA: Mono unsaturated Fatty Acids; SFA: Saturated fatty acids; PFA: Poly unsaturated fatty acid. The bold p values have passed the Benjamini–Hochberg False Discovery Threshold at p<0.05.

**Table 4:** Comparing age, sex, and race adjusted mean of dietary components by presence of triglycerides criterion

| **Variables** | **Triglyceride criterion** | | |
| --- | --- | --- | --- |
|  | **Absence** | **Presence** | **P value** |
| **Carbohydrate (gm)** | 257.2756 | 269.6702 | 1.0E-07 |
| **Total sugars (gm)** | 111.903 | 119.6186 | 1.0E-06 |
| **MFA 16:1 (Hexadecenoic) (gm)** | 1.1761 | 1.2408 | 4.3E-05 |
| **SFA 18:0 (Octadecanoic) (gm)** | 6.278 | 6.6653 | 4.5E-05 |
| **Lutein + zeaxanthin (mcg)** | 1550.51 | 1315.23 | 2.9E-04 |
| **Total saturated fatty acids (gm)** | 24.6358 | 25.8201 | 1.0E-03 |
| **Folic acid (mcg)** | 182.47 | 192.46 | 2.0E-03 |
| **SFA 16:0 (Hexadecanoic) (gm)** | 13.5214 | 14.0993 | 2.0E-03 |
| **PFA 22:6 (Docosahexaenoic) (gm)** | 0.0897 | 0.0784 | 2.0E-03 |
| **Vitamin K (mcg)** | 109.693 | 93.5828 | 5.0E-03 |
| **SFA 14:0 (Tetradecanoic) (gm)** | 1.978 | 2.0874 | 5.0E-03 |
| **Sodium (mg)** | 3449.7 | 3553.61 | 6.0E-03 |
| **Vitamin E as alpha-tocopherol (mg)** | 7.6169 | 7.2617 | 8.0E-03 |
| **Cholesterol (mg)** | 292.15 | 304.25 | 9.0E-03 |
| **Added vitamin B12 (mcg)** | 0.8909 | 0.7644 | 1.0E-02 |
| **Zinc (mg)** | 11.471 | 12.0157 | 1.2E-02 |
| **PFA 22:5 (Docosapentaenoic) (gm)** | 0.0252 | 0.0234 | 1.2E-02 |
| **PFA 20:5 (Eicosapentaenoic) (gm)** | 0.047 | 0.0413 | 2.7E-02 |
| **Folate, DFE (mcg)** | 531.21 | 544.98 | 3.9E-02 |
| **Protein (gm)** | 82.0206 | 83.7472 | 4.7E-02 |
| **Phosphorus (mg)** | 1318.63 | 1346.01 | 4.7E-02 |
| **Total choline (mg)** | 330.8623 | 338.8867 | 5.1E-02 |
| **SFA 4:0 (Butanoic) (gm)** | 0.4821 | 0.5042 | 5.3E-02 |
| **Beta-carotene (mcg)** | 2195.63 | 1998.33 | 5.7E-02 |
| **Added alpha-tocopherol (Vitamin E) (mg)** | 0.4529 | 0.3553 | 5.9E-02 |
| **Total fat (gm)** | 77.3643 | 79.1151 | 8.6E-02 |
| **Iron (mg)** | 15.0196 | 15.3202 | 8.8E-02 |
| **Calcium (mg)** | 897.75 | 916.45 | 8.9E-02 |
| **Total monounsaturated fatty acids (gm)** | 28.3721 | 29.0167 | 9.0E-02 |
| **Thiamin (Vitamin B1) (mg)** | 1.5941 | 1.6276 | 9.9E-02 |
| **Theobromine (mg)** | 31.23 | 33.83 | 1.1E-01 |
| **MFA 18:1 (Octadecenoic) (gm)** | 26.4986 | 27.0785 | 1.1E-01 |
| **SFA 6:0 (Hexanoic) (gm)** | 0.2675 | 0.2778 | 1.2E-01 |
| **SFA 12:0 (Dodecanoic) (gm)** | 0.6783 | 0.7121 | 1.4E-01 |
| **MFA 22:1 (Docosenoic) (gm)** | 0.033 | 0.0296 | 1.5E-01 |
| **Magnesium (mg)** | 297.28 | 293.17 | 1.5E-01 |
| **SFA 10:0 (Decanoic) (gm)** | 0.4027 | 0.4159 | 1.5E-01 |
| **Total folate (mcg)** | 403.3 | 410.09 | 1.6E-01 |
| **Dietary fiber (gm)** | 16.8235 | 16.6 | 2.1E-01 |
| **PFA 20:4 (Eicosatetraenoic) (gm)** | 0.1547 | 0.1579 | 2.1E-01 |
| **Alpha-carotene (mcg)** | 424.64 | 386.88 | 2.2E-01 |
| **PFA 18:3 (Octadecatrienoic) (gm)** | 1.5792 | 1.5491 | 2.4E-01 |
| **SFA 8:0 (Octanoic) (gm)** | 0.2206 | 0.227 | 2.5E-01 |
| **Vitamin C (mg)** | 93.195 | 91.0096 | 2.5E-01 |
| **Selenium (mcg)** | 112.7641 | 114.2248 | 2.6E-01 |
| **Food folate (mcg)** | 220.86 | 217.64 | 3.2E-01 |
| **Total polyunsaturated fatty acids (gm)** | 17.6699 | 17.4293 | 3.7E-01 |
| **Riboflavin (Vitamin B2) (mg)** | 2.0164 | 2.0367 | 3.8E-01 |
| **PFA 18:2 (Octadecadienoic) (gm)** | 15.5673 | 15.3645 | 4.0E-01 |
| **Beta-cryptoxanthin (mcg)** | 110.21 | 113.99 | 4.2E-01 |
| **Caffeine (mg)** | 136.75 | 139.67 | 5.3E-01 |
| **Retinol (mcg)** | 381.83 | 389.51 | 5.6E-01 |
| **MFA 20:1 (Eicosenoic) (gm)** | 0.2663 | 0.2633 | 5.9E-01 |
| **Vitamin A, RAE (mcg)** | 586.99 | 576.84 | 6.2E-01 |
| **Vitamin B6 (mg)** | 2.0323 | 2.0206 | 6.5E-01 |
| **Potassium (mg)** | 2643.77 | 2655.3 | 6.7E-01 |
| **PFA 18:4 (Octadecatetraenoic) (gm)** | 0.01404 | 0.01416 | 8.7E-01 |
| **Vitamin B12 (mcg)** | 5.0798 | 5.1037 | 8.7E-01 |
| **Niacin (mg)** | 24.9873 | 25.033 | 8.8E-01 |
| **Alcohol (gm)** | 9.6649 | 9.7263 | 9.2E-01 |
| **Lycopene (mcg)** | 5107.76 | 5116.52 | 9.7E-01 |
| **Copper (mg)** | 1.3165 | 1.3167 | 9.9E-01 |

MFA: Mono unsaturated Fatty Acids; SFA: Saturated fatty acids; PFA: Poly unsaturated fatty acid. The bold p values have passed the Benjamini–Hochberg False Discovery Threshold at p<0.05.

**Table 5:** Comparing age, sex, and race adjusted mean of dietary components by presence of waist criteria

| **Variables** | **Waist criteria** | | |
| --- | --- | --- | --- |
|  | **Absence** | **Presence** | **p value** |
| **MFA 16:1 (Hexadecenoic) (gm)** | 1.1478 | 1.2514 | 5.0E-10 |
| **SFA 18:0 (Octadecanoic) (gm)** | 6.1429 | 6.6933 | 2.0E-08 |
| **Total saturated fatty acids (gm)** | 24.1631 | 25.9758 | 1.0E-07 |
| **SFA 16:0 (Hexadecanoic) (gm)** | 13.2581 | 14.2058 | 1.0E-07 |
| **PFA 20:4 (Eicosatetraenoic) (gm)** | 0.1499 | 0.1626 | 1.0E-07 |
| **Cholesterol (mg)** | 285.63 | 308.96 | 5.0E-07 |
| **Magnesium (mg)** | 302.58 | 289.45 | 9.0E-07 |
| **Total fat (gm)** | 75.744 | 80.2983 | 1.0E-06 |
| **Vitamin C (mg)** | 96.9668 | 87.8594 | 1.0E-06 |
| **Sodium (mg)** | 3417.45 | 3581.76 | 1.0E-06 |
| **Dietary fiber (gm)** | 17.2262 | 16.1881 | 1.0E-05 |
| **Vitamin B6 (mg)** | 2.0808 | 1.9764 | 1.2E-05 |
| **Total monounsaturated fatty acids (gm)** | 27.8009 | 29.3818 | 1.6E-05 |
| **Caffeine (mg)** | 128.08 | 146.48 | 1.8E-05 |
| **SFA 14:0 (Tetradecanoic) (gm)** | 1.9459 | 2.0912 | 2.0E-05 |
| **Added vitamin B12 (mcg)** | 0.9389 | 0.7301 | 2.4E-05 |
| **MFA 18:1 (Octadecenoic) (gm)** | 25.9749 | 27.4112 | 3.0E-05 |
| **Alcohol (gm)** | 11.1885 | 8.6628 | 1.2E-04 |
| **Beta-carotene (mcg)** | 2357.21 | 1919.57 | 1.5E-04 |
| **Food folate (mcg)** | 224.69 | 214.86 | 3.0E-04 |
| **Vitamin E as alpha-tocopherol (mg)** | 7.6991 | 7.2617 | 1.0E-03 |
| **Vitamin A, RAE (mcg)** | 599.86 | 566.4 | 1.0E-03 |
| **Beta-cryptoxanthin (mcg)** | 118.56 | 105.11 | 1.0E-03 |
| **Lutein + zeaxanthin (mcg)** | 1599.89 | 1343.88 | 1.0E-03 |
| **Total folate (mcg)** | 413.66 | 397.56 | 1.0E-03 |
| **Added alpha-tocopherol (Vitamin E) (mg)** | 0.4976 | 0.3167 | 3.0E-03 |
| **Folate, DFE (mcg)** | 546.15 | 525.61 | 3.0E-03 |
| **PFA 18:2 (Octadecadienoic) (gm)** | 15.1712 | 15.861 | 3.0E-03 |
| **Total polyunsaturated fatty acids (gm)** | 17.239 | 17.9809 | 4.0E-03 |
| **Potassium (mg)** | 2678.61 | 2619.8 | 4.0E-03 |
| **PFA 22:6 (Docosahexaenoic) (gm)** | 0.0916 | 0.082 | 4.0E-03 |
| **SFA 10:0 (Decanoic) (gm)** | 0.3966 | 0.4181 | 6.0E-03 |
| **MFA 20:1 (Eicosenoic) (gm)** | 0.2582 | 0.2729 | 7.0E-03 |
| **SFA 4:0 (Butanoic) (gm)** | 0.476 | 0.5021 | 1.1E-02 |
| **PFA 20:5 (Eicosapentaenoic) (gm)** | 0.0487 | 0.0427 | 1.6E-02 |
| **SFA 6:0 (Hexanoic) (gm)** | 0.2641 | 0.2778 | 2.0E-02 |
| **Vitamin K (mcg)** | 111.5004 | 97.0012 | 2.7E-02 |
| **Alpha-carotene (mcg)** | 448.43 | 380.35 | 2.9E-02 |
| **Protein (gm)** | 82.0235 | 83.4953 | 4.0E-02 |
| **SFA 12:0 (Dodecanoic) (gm)** | 0.6721 | 0.7187 | 4.3E-02 |
| **Niacin (mg)** | 25.2868 | 24.7816 | 6.5E-02 |
| **SFA 8:0 (Octanoic) (gm)** | 0.2192 | 0.2283 | 6.6E-02 |
| **Folic acid (mcg)** | 189.01 | 182.71 | 7.1E-02 |
| **Total choline (mg)** | 331.9913 | 337.7915 | 9.4E-02 |
| **Carbohydrate (gm)** | 264.1307 | 260.4294 | 1.0E-01 |
| **Iron (mg)** | 15.2135 | 15.0006 | 1.1E-01 |
| **PFA 18:3 (Octadecatrienoic) (gm)** | 1.5477 | 1.5908 | 1.2E-01 |
| **Total sugars (gm)** | 116.1615 | 113.997 | 1.3E-01 |
| **Thiamin (Vitamin B1) (mg)** | 1.6197 | 1.5925 | 1.6E-01 |
| **Riboflavin (Vitamin B2) (mg)** | 2.0342 | 2.0076 | 1.7E-01 |
| **Copper (mg)** | 1.3317 | 1.3031 | 1.9E-01 |
| **Theobromine (mg)** | 33.73 | 31.27 | 1.9E-01 |
| **Zinc (mg)** | 11.5519 | 11.7892 | 2.0E-01 |
| **Phosphorus (mg)** | 1322.98 | 1337.32 | 2.0E-01 |
| **MFA 22:1 (Docosenoic) (gm)** | 0.0312 | 0.0333 | 3.6E-01 |
| **Vitamin B12 (mcg)** | 5.0269 | 5.1488 | 4.0E-01 |
| **Selenium (mcg)** | 113.1441 | 114.0482 | 4.1E-01 |
| **PFA 18:4 (Octadecatetraenoic) (gm)** | 0.01403 | 0.01454 | 5.0E-01 |
| **Retinol (mcg)** | 379.88 | 386.3 | 5.3E-01 |
| **Lycopene (mcg)** | 5100.53 | 5040.31 | 7.6E-01 |
| **Calcium (mg)** | 904.24 | 902.55 | 8.5E-01 |
| **PFA 22:5 (Docosapentaenoic) (gm)** | 0.0245 | 0.0247 | 8.1E+00 |

MFA: Mono unsaturated Fatty Acids; SFA: Saturated fatty acids; PFA: Poly unsaturated fatty acid. The bold p values have passed the Benjamini–Hochberg False Discovery Threshold at p<0.05.
